# Bubble Sensors for Temperature Measurements through a Colorimetric Approach

**DOI:** 10.3390/s24041278

**Published:** 2024-02-17

**Authors:** Carlo Trigona, Sara Panebianco, Rosaria Galvagno, Anna Maria Gueli

**Affiliations:** 1 Department of Electrical Electronic and Computer Engineering, University of Catania, Viale Andrea Doria 6, 95125 Catania, Italy; sara.panebianco@unict.it; 2 Department of Physics and Astronomy “Ettore Majorana”, University of Catania, Via S. Sofia 64, 95123 Catania, Italy; anna.gueli@unict.it

**Keywords:** temperature sensor, thermochromic paint, color characterization, spectral reflectance factor, spectroradiometry

## Abstract

This paper introduces an innovative sensor utilizing bubbles coated with thermochromic paint, aiming to facilitate temperature measurements in challenging-to-reach locations without the requirement of an external power source. The research conducted is innovative in terms of both methodology and application. The characterization of the thermochromic properties of paints was, in fact, performed using spectroradiometric measurements by selecting a temperature range useful for applications in various fields including preventive conservation. The study encompasses two main objectives: (1) analyzing the color characteristics of thermochromic paint and plastic resin that forms the bubbles, and (2) assessing a temperature sensor comprising a thermochromic paint-coated bubble subjected to temperature variations. The thermochromic paint exhibits reversible color modifications in response to temperature changes, making it an ideal candidate for applications of this nature. The color characterization phase involves measurements using a spectroradiometer to compare the spectral reflectance factor (SRF%) of the colored plastic resin spread on canvas with that of the inflated bubbles. The sensor characterization entails evaluating color changes of the thermochromic paint on the bubble surface with varying temperatures. Experimental results indicate that the combination of a red (R) bubble and blue (B) thermochromic paint produces quantifiable color variations suitable for the proposed applications, whereas the alternative combination under examination, namely a blue bubble and red thermochromic paint, yields less accurate results. Considering that for both thermochromic paints the color change temperature is 35 °C, it is possible to see how, for B bubble with R thermochromic paint, the chromatic coordinates change value: C* = 3.14 ± 0.14 and *h* = 289.54 ± 11.58 at room temperature, while C* = 2.96 ± 0.12 and *h* = 304.20 ± 12.17 at 35 °C. The same is true for R bubble with B thermochromic paint where C* = 25.31 ± 1.01 and h* = 285.05 ± 11.40 at room temperature, while C* = 20.87 ± 0.85 and *h* = 288.37 ± 11.53 at 35 °C. The study demonstrates the potential of the approach and suggests further investigations into reproducibility and expanded color combinations. The results provide a promising basis for future improvements in temperature monitoring with thermochromic bubble sensors.

## 1. Introduction

In various fields, from industrial applications to everyday scenarios, the objective is to monitor different physical parameters, including temperature, humidity, vibration, and sometimes even radioactivity. Temperature, in particular, stands out as one of the most frequently monitored parameters across various sectors, especially within industrial contexts [1]. Traditional electronic sensors, such as CMOS sensors integrated with conditioning circuits [2] representing working ranges in temperatures from −55 °C to 120 °C with a sensitivity of 1100 ppm/V and an accuracy of ±0.4 °C; graphene sensors with a temperature range between room temperature and 80 °C, a bi-layer thermal sensitivity equal to 7.33 mL/s, and a resolution of 1.17 mL/s [3]; or carbon fiber-based sensors with a resistivity at 20 °C equal to (1.73 ± 0.08) × 10^4^. [4]; and MOS tunnelling diode solutions, with a temperature range of up to 250 °C [5], are extensively employed due to their cost effectiveness and versatility [6,7]. However, these sensors may face challenges when exposed to harmful measurement environments or electromagnetic interferences from various sources [8]. Additionally, many of these sensors are wireless, and despite their high performance, they are constrained by the power supply needed for operation. Nowadays, semiconductor temperature sensors are being developed in the form of p-n junction diode structures with a temperature range from 20 °C to 600 °C and a sensitivity equal to 3.5 mV/°C [9], and in the form of Schottky diodes with a temperature range from −65 °C to 85 °C [10,11]. Understandably, microelectronic sensors are being produced so widely because they have advantages in terms of measurement accuracy over wide ranges of temperature and also if there is the influence of other physical quantities, such as pressure and humidity. And in addition, they have a stable repeatability of output characteristics.

In the current landscape, research efforts aim to develop novel sensors and measurement solutions for monitoring parameters in problematic locations, such as the roof of a church or museum, where temperature regulation is crucial for preserving paintings and artworks. Importantly, traditional approaches requiring connection cables, batteries, and circuitry are impractical for such sites. This paper contributes to the advancement of the field by introducing a novel sensor based on a bubble and a thermochromic color-changing method for temperature measurements. This innovative solution, presented for the first time in the literature, has several attractive features, including low cost, battery-free operation, and easy monitoring, even at high heights, due to its light weight. Such sensors can achieve greater heights, especially when filled with specific gases. The colorimetric readout based on thermochromic material enables both optical and quantitative change assessment without the need for additional external circuitry [12].

Chromic phenomena involve changes in the color of an object due to modifications in the absorption, reflection, or refraction spectrum [13]. In recent years, chromic materials have found applications in various devices, such as smart windows for regulating solar energy [14], phase change energy storage materials [15], and wearable thermochromic sensors for portable electronics [15,16,17]. The unique functionality of chromic materials allows for user-friendly visual detection and reversible fast responses through coloration under specific parameters when integrated with other applications [18].

Thermochromism, a phenomenon in which certain dyes change color reversibly with temperature variations, involves materials like liquid crystals, leuco dyes, and water-based acrylic polymers [19]. Liquid crystals, arranged in planes with specific orientations, reflect light at wavelengths, and changes in temperature modify the reflection wavelength, causing a color change. Leuco dyes can transition from colored to colorless states due to structural changes in their molecules caused by temperature changes [20]. Acrylic polymers, widely used in industrial environments, exhibit color changes influenced by temperature variations [21].

Considering the limitations of some materials, such as the high cost and low color density of liquid crystals [22] and the toxicity of leuco dyes [23], this study opts for a water-based thermochromic acrylic polymer paint. This choice ensures easy application, non-toxicity, environmental safety, and resistance to weather conditions while maintaining the thermochromic effect [24].

With the aim of developing the temperature sensor, the study is divided into two parts. The first part focuses on color characterization, comprising the study of color differences between the spread on canvas and the color when the plastic resin is inflated to form the bubble. The second part concerns the characterization of the sensor by studying the color changes of the thermochromic paint covering the bubble as the temperature changes. The estimation of color variation is accomplished by evaluating the spectral reflectance factor (SRF%) and the colorimetric coordinates in the CIELAB color space [25].

## 2. Materials and Methods

To achieve the objectives of this study, bubbles and thermochromic paints derived from a water-based acrylic polymer composition were utilized. Regarding the paint, it is widely chosen for its attributes favoring rapid drying, water solubility, and versatility on various surfaces. It comprises three main components: a binder responsible for paint quality, a pigment, and a vehicle, which in this case was water, thus determining color saturation upon application. Specifically, the paint used was a polyurethane acrylic categorized as an organic polymer [22]. Polymer bonds consist of hard and soft segments that are resistant to different chemical solvents due to the urethane group, providing strength to the structure through hydrogen bonds. Environmental changes, especially in temperature, lead to significant modifications in the soft bonds’ chemical composition, resulting in changes in molecular weight and solubility in organic solvents, possibly due to crosslinking and oxidation [24]. This leads to modifications in surface morphology and visible color variations, making these molecular changes responsible for the observed color change in the paint.

For the experimental work, two different hues of thermochromic paints were employed: blue and red. The transition temperature of these products, as provided in their technical data sheet, was approximately ~35 °C, facilitating fast drying times (around 30 s) at room temperature. The bubbles used were gummy, colored epoxy plastic resin placed on a straw and inflated to create bubbles of variable size. Considering that bubble thickness can affect the color, the bubbles were obtained using the same amount of material, inflated by the same operator and for the same time intervals. The bubbles obtained were almost spherical in shape with an average diameter of 10 cm.

As mentioned in the introduction, the study is divided into two parts: color characterization of the thermochromic paint and resin spread on canvas, and color characterization of the sensor consisting of the bubble on which the paint was spread. Color characterization was conducted using a spectroradiometer, specifically the Konica Minolta CS-1000A (Konica Minolta, Tokyo, Japan), for non-contact measurements of objects or light sources. The spectroradiometer was remotely controlled and connected to a computer with the CS-S1w software installed, allowing for the setting of instrument measurement parameters, acquisition, display, and registration of measured quantities.

**Figure 1:**
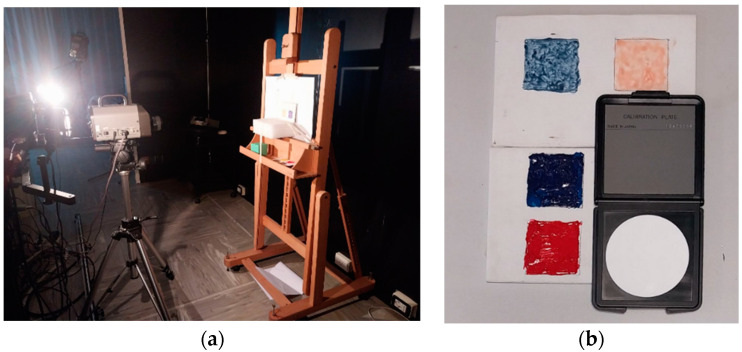
(**a**) Experimental setup for the first measurement step “color characterization” of plastic resin and paint on canvas. (**b**) Colored plastic resin (lower canvas) and thermochromic paints (upper canvas) spread on canvas, along with the white calibration plate of the spectroradiometer.

The experimental setup for the first part of the study is shown in Figure 1a, with measurements conducted on homogeneous areas of the resin and thermochromic paint applied to the canvas. Measurements were made taking care that the measuring area of the instrument (a 16 mm diameter at a distance of 60 cm) corresponded with a homogeneous paint application area. The samples used for measurements are shown in Figure 1b. Two halogen light sources were fixed to a holder, projecting beams symmetrically at 45° angles with respect to the normal direction at the studied surface (2 × 45°/0° illumination/observation geometry), following CIE recommendations for color measurements [25]. The lamps and the spectroradiometer were placed 60 cm from the sample, with reflected radiation detected along the normal direction between the object and the spectroradiometer. To control the heat produced by the halogen lamps, temperature measurements were taken on the samples using both a homemade thermocouple and a thermohygrometer (Beurer, Ulm, Germany, HM16). As measurements were conducted in an air-conditioned environment, the temperature difference measured from the ambient temperature was less than 1 °C, ensuring that the emitted heat did not impact the color of the measured samples.

**Figure 2:**
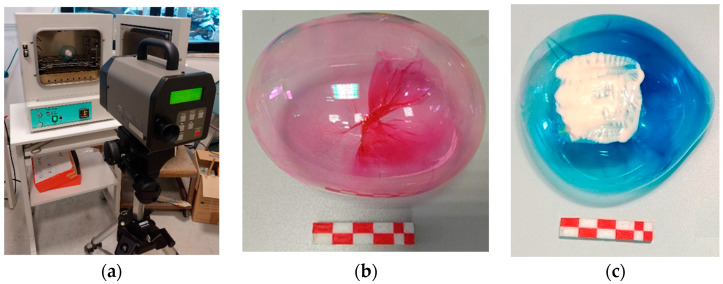
(**a**) Experimental setup for the second measurement step “sensor characterization” of bubbles in the oven. (**b**) Example of red epoxy plastic resin inflated as a bubble. (**c**) Example of a bubble sensor in one of two hue combinations used: B bubble and R thermochromic paint.

The second part of the study is related to sensor characterization, using the setup shown in Figure 2a. Colored plastic resin was employed to create bubbles in two different hues: red and blue. The bubbles were combined with two thermochromic paints, also in red and blue, according to the combinations in Table 1.

Considering the RGB additive synthesis, two combinations of bubble and thermochromic paints were chosen; the first involving a blue bubble with red thermochromic paint spread, and the second involving a red bubble with blue thermochromic paint spread.

An example of epoxy plastic resin inflated to form the bubble is shown in Figure 2b. Figure 2c provides an example of the complete sensor, consisting of a bubble and thermochromic paint in one of the two color combinations chosen for the study. After applying the coating material, a G^®^-Therm 015 drying oven (F.lli Galli G.& P., Milano, Italy) was employed to vary the temperature. Measurement points were placed on homogeneous areas at the center of squares formed by thermochromic paints. The resistance of the bubbles to temperature imposed choices in terms of values and range. Measurements were taken at fixed temperatures of 20 °C (room temperature), 35 °C, 45 °C, and 55 °C. These temperature values were assessed inside the oven using a thermocouple.

Spectroradiometer measurements were made by placing it at the same distance as the first step, i.e., 60 cm from the studied samples, with a light source with an emission spectrum relative to illuminant A. The emission spectrum of the source was measured with the spectroradiometer in light source mode. The 10° standard observer was selected in the acquisition settings to acquire the colorimetric parameters for object color mode. In this last measurement stage, the studied samples were measured directly inside the oven to ensure the reproducibility of the measurements, mainly with regard to the positioning of the measuring points.

### Elaboration of Measured Data

The spectral reflectance factor (SRF%) behavior in terms of a graphical representation of the spectral response at different wavelengths of the visible electromagnetic spectrum was used to measure the optical behavior of the materials under analysis. The CIELAB (L*, a*, b*) color coordinates, derived from the SRF behavior, were utilized for color specification through spectroradiometer measurements. In this color space, L* designates the achromatic axis, while a* and b* correspond to the color axes. The L* parameter quantifies the lightness of a given hue. The a* coordinate defines the color along the red–green axis, and b* determines the color along the yellow–blue axis.

To highlight chromatic variations, it is recommended to employ polar coordinates within the CIELAB (L*, C*, *h*) color space [25]. C* specifies chroma, and *h* represents the hue angle as an angular measurement, both derived from CIELAB (L*, a*, b*) using the following formulas:

(1) $$C^{*}=\sqrt{{\left(a^{*}\right)}^{2}+{\left(b^{*}\right)}^{2}}$$

(2) $$h=arctg\left(b^{*}/a^{*}\right)$$

The experimental uncertainty is calculated as the sum in quadrature of the uncertainty due to the instrument response (1% of the measurement) and from the instrument scale adjustment (white calibration plate for the spectroradiometer), plus the type-A uncertainty associated with repeated measurements (3 measurements for each measured point) [26].

## 3. Results and Discussion

This section presents the results obtained for each type of analysis. The Section 3.1 details the measurements of the color coordinates for both the resin spread on canvas and the inflated bubbles. Measurements for the latter were also conducted at different temperatures. The Section 3.2 reports the analysis of the optical behaviour of the materials under examination through the SRF (%) trend. The Section 3.3 examines the development of the color co-ordinates of the bubbles with the paint layer as the temperature changes, with the aim of assessing its potential as a temperature sensor.

### 3.1. Color Specification

The initial-phase outcomes, involving color specification of plastic resin on canvas and inflated bubbles, are presented in Table 2 through color coordinate values in CIELAB spaces (L*, a*, b*) and (L*, C*, *h*). Computation of C* and *h* values is executed using Equations (1) and (2), respectively, with uncertainties calculated in the manner mentioned previously. In this preliminary stage, adjustments to the lightness scale were made using the white reference to assess measurement uncertainties [26].

For sensor characterization, bubbles coated with thermochromic paint in R and B colors were employed, following the RGB model outlined in Table 1. These sensors were subjected to oven temperatures varying from 35 to 55 degrees in 10-degree increments. Results of color specification measurements for the sensor are presented in Table 3, detailing color coordinates in CIELAB spaces (L*, a*, b*) and (L*, C*, *h*). Again, C* and *h* were evaluated using Equations (1) and (2), respectively.

### 3.2. Optical Characterization

Measurements were conducted to investigate the optical properties of the materials under examination, showing the relative SRF (%).

**Figure 3:**
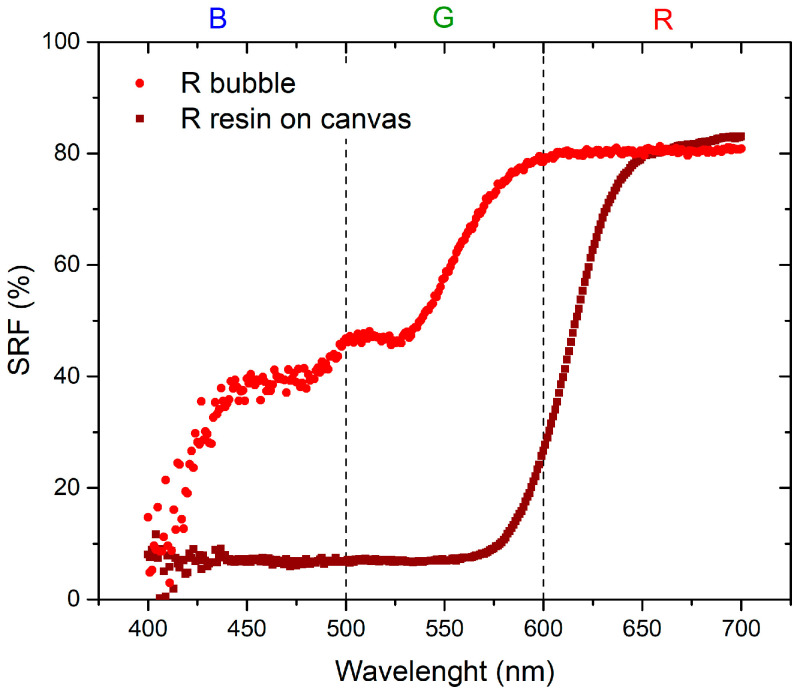
Comparison between R plastic resin spread on a canvas and inflated R bubble.

**Figure 4:**
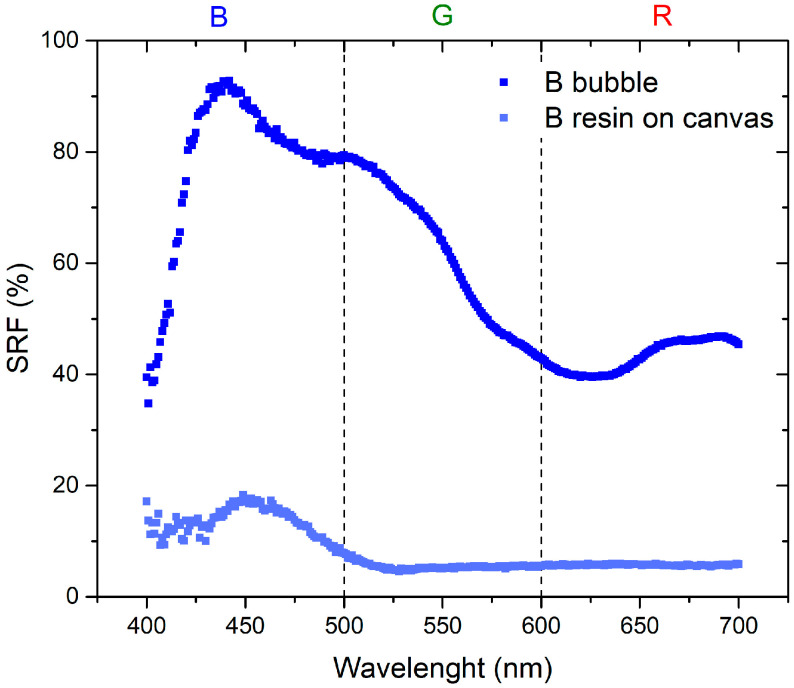
Comparison between B plastic resin painted on a canvas and inflated B bubble.

The initial set of measurements aimed to compare the optical properties of the resin applied to the canvas and the inflated bubble. Figure 3 and Figure 4 visually demonstrate that the color becomes more saturated when the resin is applied to the canvas. This phenomenon occurs because when the plastic resin is inflated to form the bubble, it tends to lose its color brightness, leading to a reduction in saturation. Specifically, in the red region, the SRF (%) exhibits minor variations, suggesting that increased transparency may result in phenomena such as refraction or diffraction, thereby affecting the reflection of light. This influence is particularly pronounced in the blue and green regions, as indicated by Figure 3.

The second set of measurements aimed to compare the optical properties of the same thermochromic paint applied to both the canvas and the inflated bubble.

**Figure 5:**
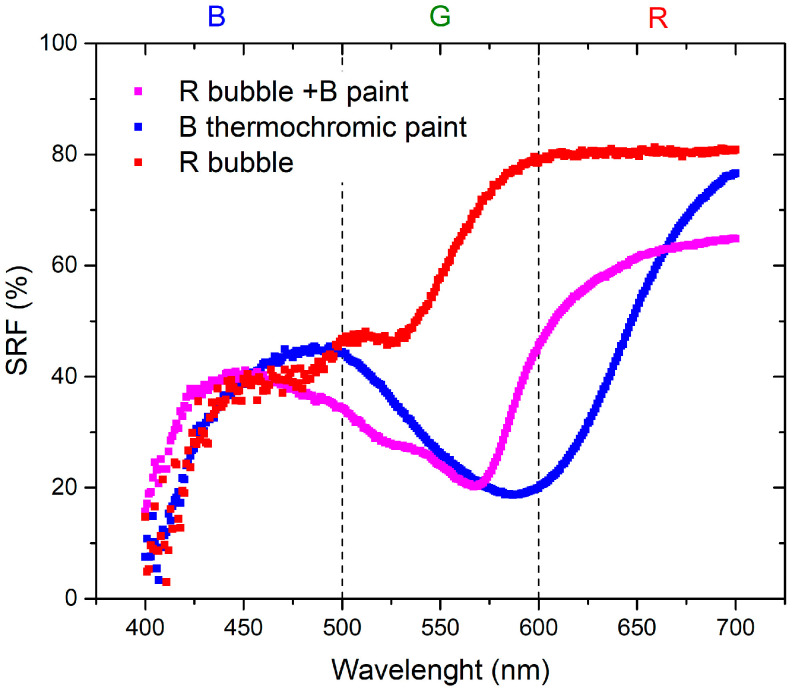
Comparison of the R bubble, B paint spread on the canvas, and R bubble with B paint coating.

**Figure 6:**
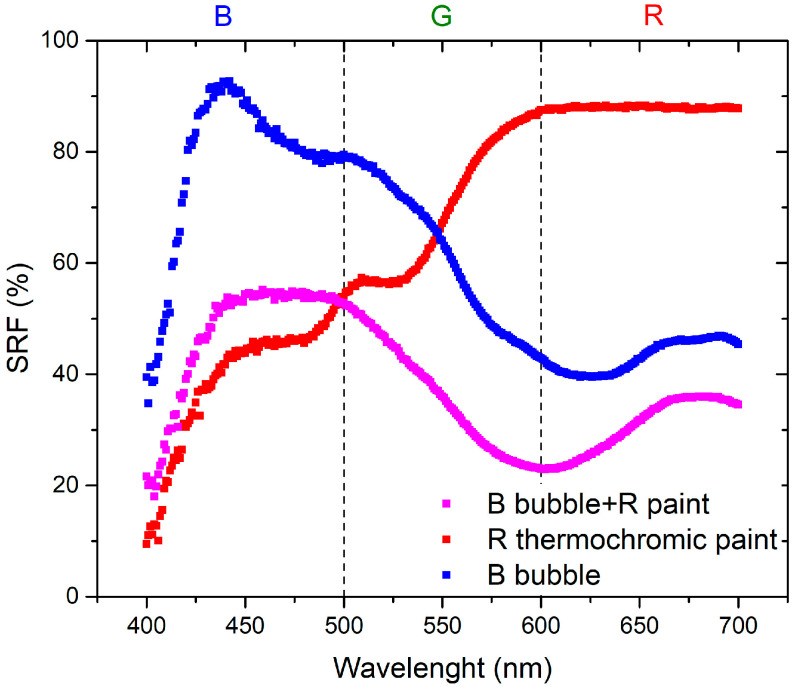
Comparison of the B bubble, R paint spread on the canvas, and B bubble with the R coating.

The variation in the SRF (%) is more pronounced in the case where the paint is applied to the bubble because the reflectance spectrum incorporates the color component of the bubble itself. This is evident in the red-hue region in Figure 5 and the blue-hue region in Figure 6. The contributions from the bubble, whether from the R or B bubble, play a predominant role in determining the SRF (%) of the combination of the bubble painted with thermochromic paint.

**Figure 7:**
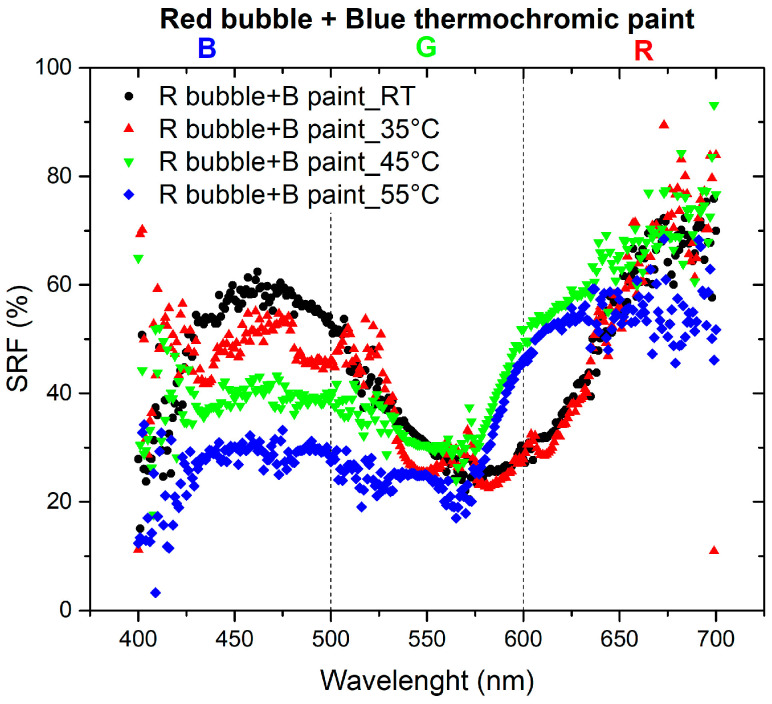
SRF (%) of R bubble with B thermochromic paint at different temperatures.

**Figure 8:**
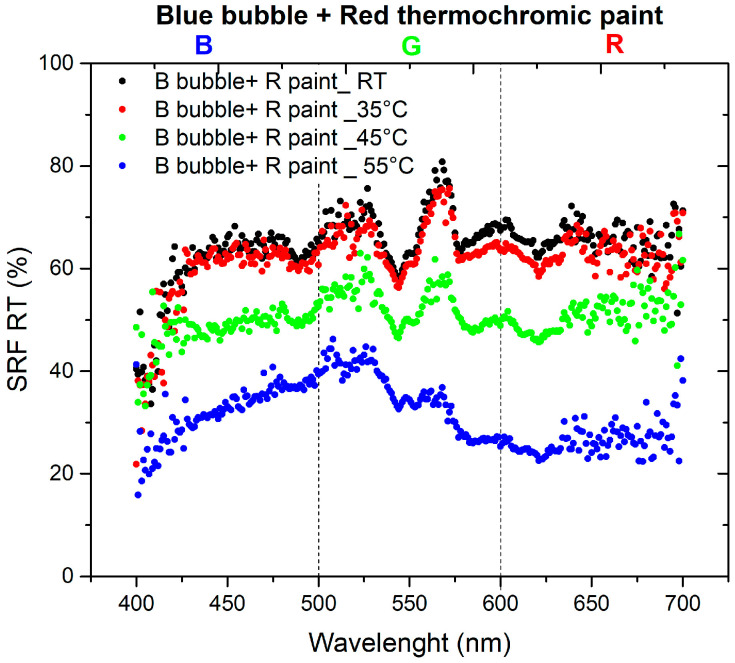
SRF (%) of B bubble with R thermochromic paint at different temperatures.

The final set of measurements aimed to investigate the optical changes of the thermochromic paint when applied to the bubble to create the sensor, observing how it responds to variations in oven temperature. To depict the color variation of the paint and the corresponding spectrum changes, the spectra of bubbles at room temperature (RT) are compared with the spectra of the paint color as the temperature varies. The SRF (%) curves are depicted in Figure 7 and Figure 8.

The materials used in this study are characterized by the combination of red and blue colors: red bubble with blue paint and blue bubble with red paint. The absence of the reflected spectral component in green implies that the resulting color corresponds to magenta [23]. As shown in the spectra presented in Figure 7, the SRF (%) of the R bubble and B paint aligns with the predicted behavior. The spectrum of the B bubble with R paint, as shown in Figure 8, should exhibit the same pattern as the R bubble with B paint. However, this is not observed because, as illustrated in Figure 4, the R paint possesses spectral components within its range and others. Additionally, it appears that during inflation, the B color of the bubbles tends to lose homogeneity, resulting in decreased saturation, behavior that becomes less pronounced in the case of the R bubble, as can be seen in the SRF (%) curves in Figure 3 and Figure 4.

To assess the change between measurements of color distribution on canvas and the color of the inflated bubble, normalized derivatives of the normalized SRF (%) versus wavelengths, ranging from 0 to 1, are employed.

**Figure 9:**
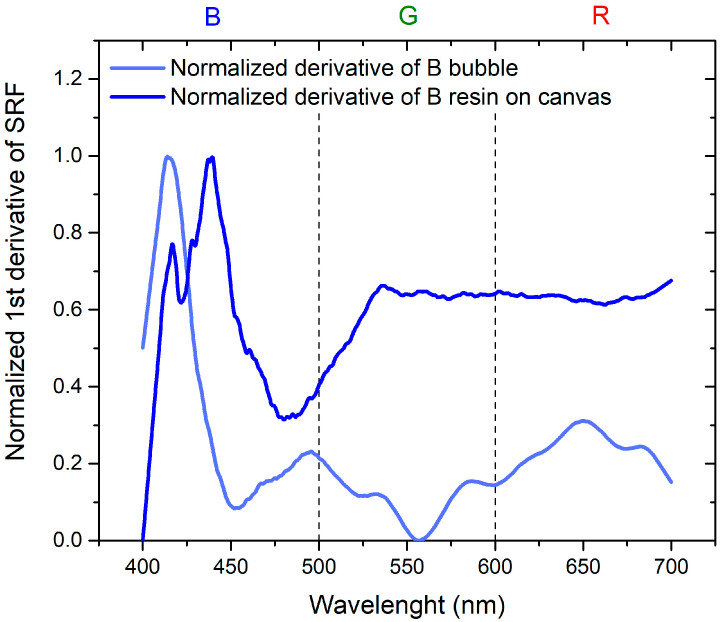
Normalized first derivative of SRF for B bubble and B plastic resin on canvas.

**Figure 10:**
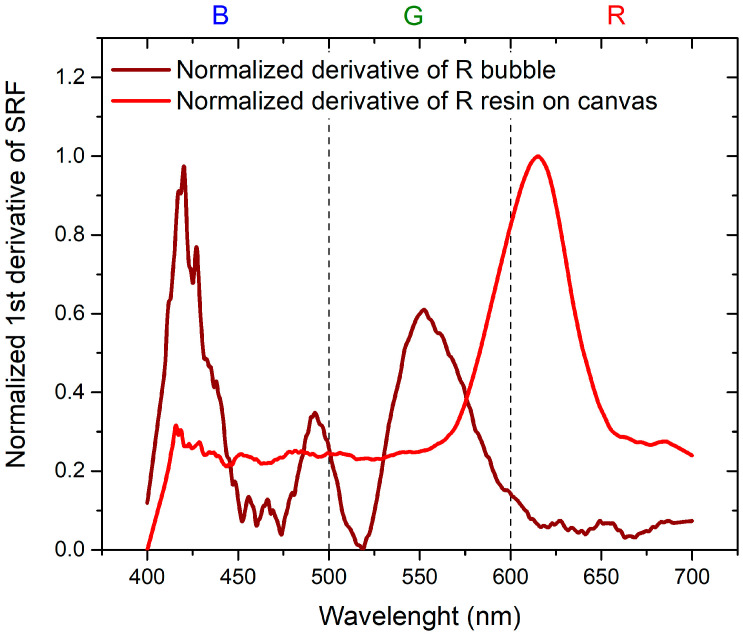
Normalized first derivative of SRF for R bubble and R plastic resin on canvas.

Regarding Figure 9 and Figure 10, a remarkable observation is the systematic shift toward the shorter wavelengths of the SRF (%) when switching from plastic resin on canvas to inflated bubble. Examining the characteristic peaks centered at 625 nm for red plastic resin on canvas and 450 nm for blue plastic resin on canvas, a shift in the maximum to 550 nm and 420 nm, respectively, is evident after inflation. Additionally, there is a reduction in the intensity of the SRF peak for the R bubble compared to the peak of the R plastic resin on canvas, while the intensity remains constant in the case of the B bubble.

**Figure 11:**
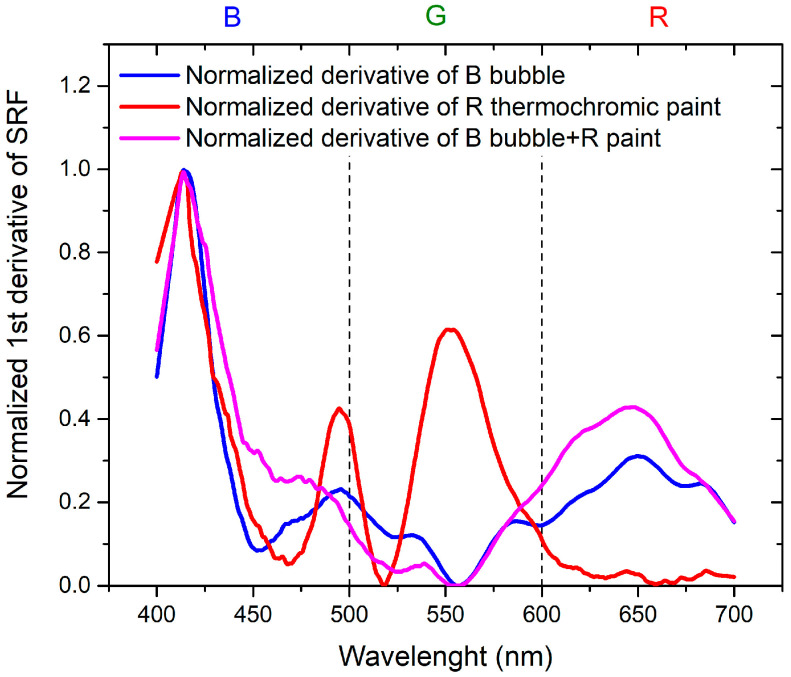
Normalized first derivative of SRF for B bubble, R thermochromic paint, and B bubble covered with R thermochromic paint.

**Figure 12:**
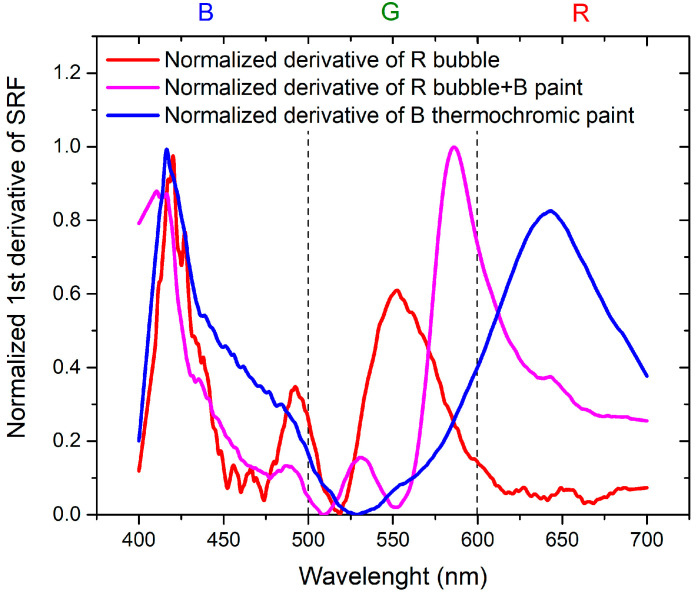
Normalized first derivative of SRF for R bubble, B thermochromic paint, and R bubble covered with B thermochromic paint.

To achieve the objectives of the study, it is essential to compare the results in order to separate the contribution of the thermochromic paint from that of the bubble. This is achieved by considering the normalized derivatives of SRF as a function of wavelength, illustrated in Figure 11 for B bubble with R thermochromic paint and in Figure 12 for R bubble and B thermochromic paint.

Regarding Figure 11, it is evident that the peak at 650 nm is attributable to the bubble, while the peak at about 500 nm is the contribution of the thermochromic paint. In the normalized derivative, the peak at 400 nm, representing the contribution of B bubble and R paint, is also present in the case of the combination of B bubble with R paint. The peak shared by the bubble and paint at 500 nm shifts to shorter wavelengths and widens in the case of the combined B bubble and R paint. The minimum at 600 nm, originating from the B bubble, disappears in the combination represented by the painted bubble. As illustrated in Figure 12, the contribution of the R bubble at 550 nm and the contribution of the B paint at 650 nm result in the combined B bubble and R paint showing a high-intensity peak at 600 nm. A distinctive component present in the sum is the peak at 530 nm, absent in both the R bubble and B paint. In the case of this sample, no significant peak shifts are observable.

**Figure 13:**
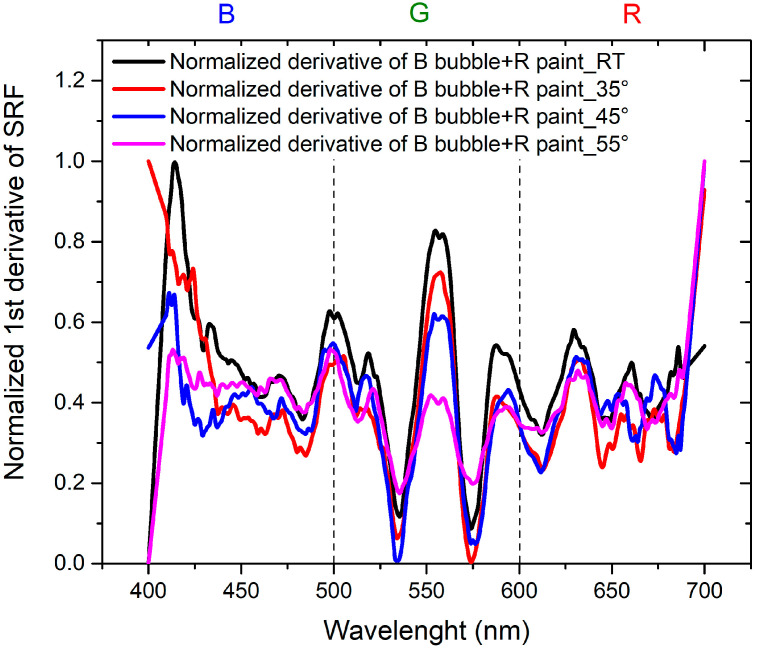
Normalized first derivative of SRF for B bubble covered with R thermochromic paint as the temperature varies.

**Figure 14:**
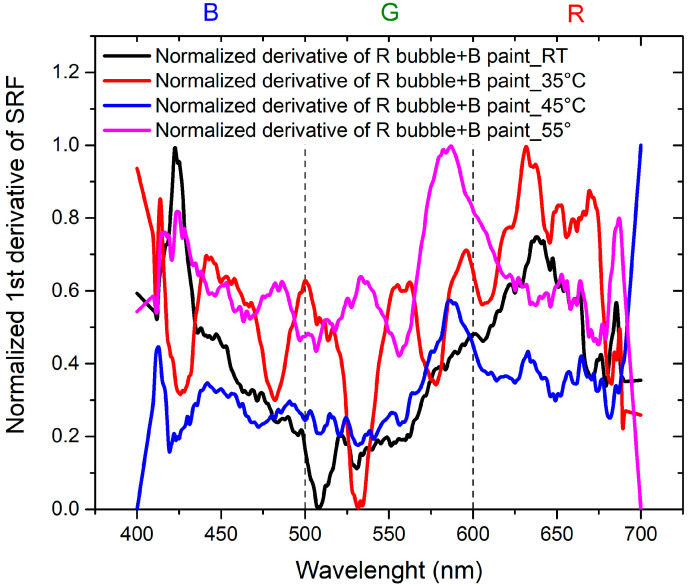
Normalized first derivative of SRF for R bubble covered with B thermochromic paint as the temperature varies.

Figure 13 illustrates the normalized derivative of the SRF (%) curves for the combination of the B bubble and R paint as the temperature varies. In this scenario, the behaviors are comparable, with the peak at room temperature (RT) being predominant compared to the peaks at higher temperatures, whose contributions gradually decrease. Figure 14 shows the behavior of the derivative of the normalized SRF in the case of the R bubble and B paint. In contrast to the previous case, the SRF curve with the highest values is observed at 55 °C, the highest temperature for which measurements are recorded.

The trends exhibit comparability, showing a certain homothety between the RT curve and the one at 35 °C, as well as between the curve at 45 °C and the curve at 55 °C. Curves obtained at higher temperatures reveal a component at 600 nm, which is absent in the RT curve. The minimum at 510 nm at RT shifts to longer wavelengths in the curve at 35 °C.

### 3.3. Sensor Characterization

Investigating the variations in chroma (C*) and hue angle (*h*) of the bubbles in response to temperature changes provided insights into the sensor’s potential as a temperature sensor.

**Figure 15:**
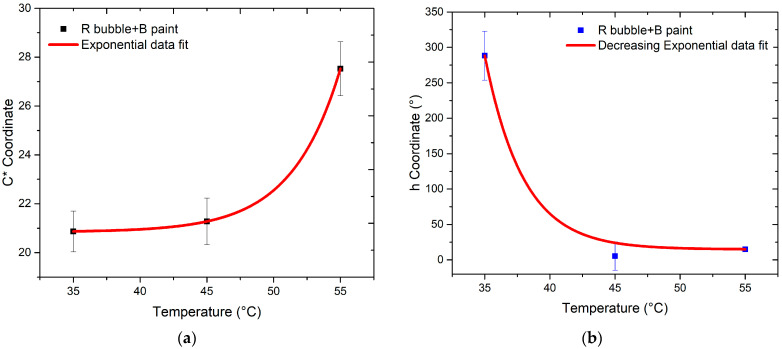
Trend of color coordinates (**a**) C* and (**b**) *h* as temperature changes for R bubble coated with B thermochromic paint.

**Figure 16:**
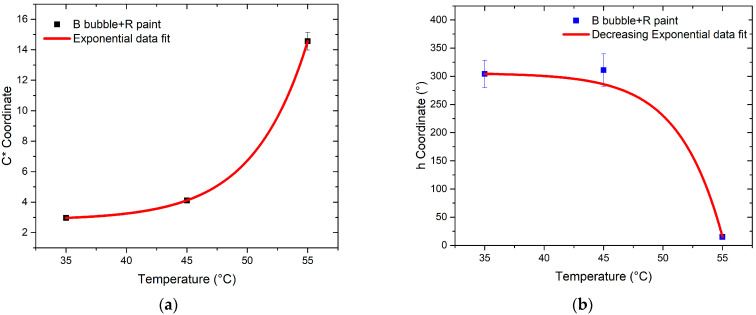
Trend of color coordinates (**a**) C* and (**b**) *h* as temperature changes for B bubble coated with R thermochromic paint.

The evaluation of color differences with changing temperatures for the R and B bubbles, respectively, is depicted in Figure 15 and Figure 16. The values of C* and *h* for temperatures exceeding 35 °C are represented. Indeed, at this temperature value, according to the technical data provided by the manufacturer, the color change in the thermochromic paint is to be expected. The trends of C* and *h* versus temperature are similar for both bubbles; in particular, the first coordinate shows a nonlinear behavior indicative of the nonlinear response of the sensor, while the second coordinate decreases as temperature increases. Since thermochromic paint undergoes a color change at 35 °C, a drastic shift is expected for this value. This is evident in the case of B thermochromic paint, for which there is a “jump” between the hue value at 35 °C and 45 °C. Moreover, in the case of R thermochromic paint, the hue value undergoes a change between 35 °C and 45 °C, but the most significant change occurs between 45 °C and 55 °C.

By performing a fit of the data, the behavior of C* values are described by an increasing exponential function in the case of both B bubble + R paint and R bubble + B paint. The function describing the data trend is as follows:

(3) $$y(x)=y_{0}+A\;exp\left(M\;x\right)$$

The trend of h-values is described by Equation (4):

(4) $$y(x)=y_{0}+A\;exp\left(-M\;x\right)$$

In contrast, the graph of h shows a decreasing exponential trend for both samples.

## 4. Conclusions

The main objective of the presented study is the characterization of an innovative temperature sensor consisting of a bubble, obtained from a specific plastic resin, on which a thermochromic paint is applied. The potential sensor requires no power supply and could therefore be used in impractical places. Thermochromic paint, with its unique feature of changing color with temperature variation, serves as the focal point. Two sections can be distinguished in the study: the first is concerned with the investigation of the color characteristics of both the thermochromic paint and the plastic resin, while the second is focused on the characterization of the potential sensor constituted by the bubble painted with the thermochromic paint.

The first part of the investigation involves a comparison between colors of the resin on canvas and those when the bubbles are inflated. As for the plastic resin, as seen in Figure 3 and Figure 4, there is a variation between the color of the resin on the canvas and the bubble. This discrepancy may be attributed to inflation, causing non-uniform color distribution and saturation. Concerning the behavior of the thermochromic paint, as shown in Figure 5 and Figure 6, the difference between paint spread on canvas and on bubble depends on the bubble’s color. Different color combinations yield different results.

The second part of the investigation involves the study of a temperature sensor consisting of a bubble coated with thermochromic paint that changes color as the temperature changes. Figure 7 shows a trend of SRF in agreement with that predicted by subtractive synthesis, reflecting light in the blue and red wavelengths, with negligible SRF in the green region.

A similar result should also be yielded from the combination of blue bubble and red thermochromic paint, but the trends of the SRF spectra in Figure 8 do not show the typical trend of magenta as in the previous case. This discrepancy occurs because of the components in the blue and green regions of the red paint, as visible in Figure 5. Concerning the independence of color from temperature variation (Figure 15 and Figure 16), the C* coordinate exhibits a non-linear trend for both combinations, indicating the sensor’s non-linear response. Regarding hue angle, the trend of R bubble + B paint at 45 °C may be attributed to limited reproducibility in bubble size and resin quantity, requiring further investigation. The jump in hue value between 35 °C and 45 °C suggests that the optimal color combination for thermochromic paint and plastic resin is blue and red, respectively. In summary, the consistent color change temperature of 35 °C for both thermochromic paints is significant. For the B bubble with R thermochromic paint, chromatic coordinates exhibit variations at room temperature (C* = 3.14 ± 0.14, *h* = 289.54 ± 11.58) and at 35 °C (C* = 2.96 ± 0.12, *h* = 304.20 ± 12.17). Similarly, the R bubble with B thermochromic paint displays changes in chromatic coordinates at room temperature (C* = 25.31 ± 1.01, *h* = 285.05 ± 11.40) and at 35 °C (C* = 20.87 ± 0.85, *h* = 288.37 ± 11.53).

In conclusion, the R bubble + B paint combination exhibits quantifiable and useful color variations for the proposed applications. The same cannot be asserted for the B bubble + R paint combination, although systematic color changes at different temperatures for these samples cannot be excluded.

This work demonstrates the potential of the experimental approach, with ongoing efforts to conduct a detailed study for increased measurement statistics and exploration of other color combinations between bubbles and paint. Perspectives include extending the temperature range for measurements and establishing a procedure for consistent paint deposition on bubbles, minimizing variability. A series of measurements aimed at identifying the possible influence of humidity variations is planned.

Prospects involve exploring additional color combinations of plastic resin and thermochromic paint following the RGB model, aiming for reproducible inflation, which remains a challenge in maintaining uniform bubble size and resin quantity. A specific aim will be to obtain bubbles with the same size and thickness to minimize the influence of color intensity.

## Figures and Tables

**Table 1:** Color combinations of bubbles and thermochromic paints used for this study.

	Bubble Color	Thermochromic Paint
First combination	Red	Blue
Second combination	Blue	Red

**Table 2:** Color coordinates in the CIELAB spaces (L*, a*, b*) and (L*, C*, *h*) obtained by spectroradiometer measurements on bubble resins spread on canvas and inflated bubbles.

	L* ± ẟL*	a* ± ẟa*	b* ± ẟb*	C* ± ẟC*	*h* ± *ẟh* (*°*)
White calibration plate	97.90 ± 0.02	−0.27 ± 0.01	−0.32 ± 0.01	0.42 ± 0.01	49.87 ± 1.50
B resin on canvas	18.15 ± 0.54	4.20 ± 0.08	−32.17 ± 0.64	32.44 ± 0.97	82.60 ± 2.48
R resin on canvas	47.19 ± 1.42	55.66 ± 1.11	47.86 ± 0.96	73.41 ± 2.20	40.71 ± 1.22
B bubble	79.43 ± 3.18	−18.95 ± 0.38	−24.06 ± 0.48	30.63 ± 1.23	28.18 ± 1.13
R bubble	75.55 ± 3.02	15.01 ± 0.30	−1.44 ± 0.03	15.08 ± 0.60	354.82 ± 14.18

**Table 3:** Color coordinates in the CIELAB spaces (L*, a*, b*) and (L*, C*, *h*) assessed through spectroradiometer measurements, showing the response of sensors made from bubbles coated with thermochromic paint to variations in oven temperature.

	L* ± ẟL*	a* ± ẟa*	b* ± ẟb*	C* ± ẟC*	*h* ± *ẟh* (°)
Calibration white	97.47 ± 0.03	−0.08 ± 0.01	−0.53 ± 0.01	0.54 ± 0.02	81.51 ± 3.26
B bubble + R paint (RT)	85.66 ± 3.43	−1.14 ± 0.02	3.21 ± 0.06	3.41 ± 0.14	289.54 ± 11.58
B bubble + R paint (35 °C)	84.23 ± 3.37	−1.67 ± 0.03	2.45 ± 0.05	2.96 ± 0.12	304.20 ± 12.17
B bubble + R paint (45 °C)	76.99 ± 3.08	−3.73 ± 0.07	1.73 ± 0.03	4.12 ± 0.16	335.12 ± 13.41
B bubble + R paint (55 °C)	63.07 ± 2.52	−14.06 ± 0.28	−3.81 ± 0.08	14.57 ± 0.58	15.16 ± 0.61
R bubble + B paint (RT)	64.83 ± 2.59	6.59 ± 0.13	−24.44 ± 0.49	25.31 ± 1.01	285.05 ± 11.40
R bubble + B paint (35 °C)	64.22 ± 2.57	6.59 ± 0.0.13	−19.80 ± 0.40	20.87 ± 0.83	288.37 ± 11.53
R bubble + B paint (45 °C)	69.82 ± 2.79	21.24 ± 0.42	1.24 ± 0.02	21.28 ± 0.85	3.35 ± 0.13
R bubble + B paint (55 °C)	64.47 ± 2.58	26.59 ± 0.53	7.12 ± 0.14	27.53 ± 1.10	15.00 ± 0.60

## Data Availability

The datasets presented in this article are not readily available because the data are part of an ongoing study. Requests to access the datasets should be directed to rosaria.galvagno@phd.unict.it.
